# The Impact of Marijuana (*Cannabis sativa*) on Viability and Expression of Cardiac Markers in Human Cardiomyocytes

**DOI:** 10.5812/ijpr-168324

**Published:** 2026-05-17

**Authors:** Hossein Kargar Jahromi, Vida Khalafi, Vida Mirzaei, Faezeh Ebrahimi-Zadeh, Pouria Azami, Tara Abdolahy, Iman Razeghian-Jahromi

**Affiliations:** 1 Research Center for Noncommunicable Diseases, Jahrom University of Medical Sciences, Jahrom, Iran; 2 Student Research Committee, Jahrom University of Medical Sciences, Jahrom, Iran; 3 Department of Cardiology, Shiraz University of Medical Sciences, Shiraz, Iran; 4 Cardiovascular Research Center, Shiraz University of Medical Sciences, Shiraz, Iran

**Keywords:** Marijuana, *Cannabis sativa*, Viability, Cardiac Biomarkers, Human Cardiomyocytes

## Abstract

**Background:**

*Cannabis sativa* has been used since antiquity for medicinal, ceremonial, and agricultural purposes. Today, cannabis, commonly known as marijuana, remains the most widely used recreational drug worldwide. As its use increases, particularly among young adults, reports of adverse effects on vital organs have also increased. This trend underscores the need for a comprehensive assessment of marijuana’s effects on the cardiovascular system.

**Objectives:**

This study aimed to investigate the effects of *C. sativa* extract on the viability, proliferation, and expression of cardiac markers in human cardiomyocytes.

**Methods:**

Human cardiomyocytes (HCMs) were cultured and subsequently exposed to varying concentrations of cannabis extract. Cell viability was assessed using the MTT assay, and morphological changes were examined on days 3 and 6. Growth curves and population doubling times were determined over a 6-day period using the trypan blue exclusion method. In addition, the expression levels of cardiac-specific markers, including GATA4, troponin, and creatine kinase, were quantified using real-time polymerase chain reaction on days 1, 3, and 6.

**Results:**

No significant changes in cell morphology were observed; however, cell density was consistently higher in the treated group, particularly at early time points. Growth-curve analysis confirmed these findings, demonstrating higher cell numbers in treated cultures throughout the study period, consistent with the population doubling time data (74.79 h in the treated group vs. 80.45 h in the control group, P = 0.005). Furthermore, the treated group exhibited increased expression of cardiac-specific markers compared with the controls, most notably between day 1 and day 3 (GATA4: 7.97 ± 1.06 vs. 1.48 ± 0.64, P < 0.05; troponin: 3.52 ± 0.85 vs. 1.13 ± 0.92, P = 0.05; creatine kinase: 0.001 ± 0.0008 vs. 1.22 ± 0.90, P = 0.05).

**Conclusions:**

*Cannabis sativa* extract increased proliferation and upregulated the expression of cardiac genes in HCMs. These changes may impair normal cardiomyocyte function, which is vital for cardiovascular health. This issue is particularly important given the increasing global consumption of cannabis-derived substances, driven in part by expanding legalization.

## 1. Background

*Cannabis sativa*, commonly known as marijuana, is among the most widely used recreational substances worldwide, primarily because of its pronounced effects on the central nervous system (1). In recent years, widespread decriminalization in several countries has markedly influenced public attitudes toward cannabis, particularly among younger populations (2). The plant produces two principal bioactive constituents: cannabidiol (CBD), which is non-psychoactive, and tetrahydrocannabinol (THC), its psychoactive counterpart (3). Cannabidiol has demonstrated therapeutic potential in various cardiovascular conditions, including diabetic cardiomyopathy, ischemia-induced arrhythmias, and myocardial infarction (MI) (4-7). In contrast, THC primarily exerts its effects through modulation of the endocannabinoid system (ECS), a complex receptor network involved in regulating immune and endocrine functions, as well as blood pressure and heart rate (2). Notably, ECS receptors are minimally expressed in the hearts of healthy individuals; however, their expression is substantially altered during the progression of cardiovascular diseases (8).

Cannabinoid receptor type 1 (CB1) receptors are present in both the heart (9) and the vasculature (10, 11), whereas cannabinoid receptor type 2 (CB2) receptors are exclusively expressed in the heart (12). These receptors are implicated in multiple pathophysiological and cardiovascular processes (13-18), including the regulation of cell proliferation (19), progenitor cell differentiation, proteolytic activity, cell death, and metabolic functions in vascular and cardiac tissues (20). Evidence suggests that CB1 and CB2 receptors may exert protective effects by safeguarding cardiomyocytes from damage and modulating cardiometabolic risk factors and atherogenesis (21). Furthermore, the administration of cannabinoids such as THC and CBD induces complex hemodynamic effects, influencing heart rate and contractility and producing alternating phases of hypotension and hypertension (22, 23).

Recent studies indicate that cannabis abuse may contribute to both cardiovascular and neurological disorders (24-26). Notably, cannabis use has been associated with an elevated risk of cardiovascular mortality in young individuals and has been recognized as a risk factor for MI in the past 5 years (27, 28). Moreover, cannabis consumption increases the risk of stroke, accounting for ischemic events in 15% to 40% of cases among young patients (29). Despite these findings, the specific effects of cannabis on the cardiac system, particularly on cardiomyocytes, remain poorly understood.

## 2. Objectives

This study aimed to investigate the effects of cannabis on the biological activity of human cardiomyocytes.

## 3. Methods

### 3.1. Culture of Human Cardiomyocytes

**Figure 1.**
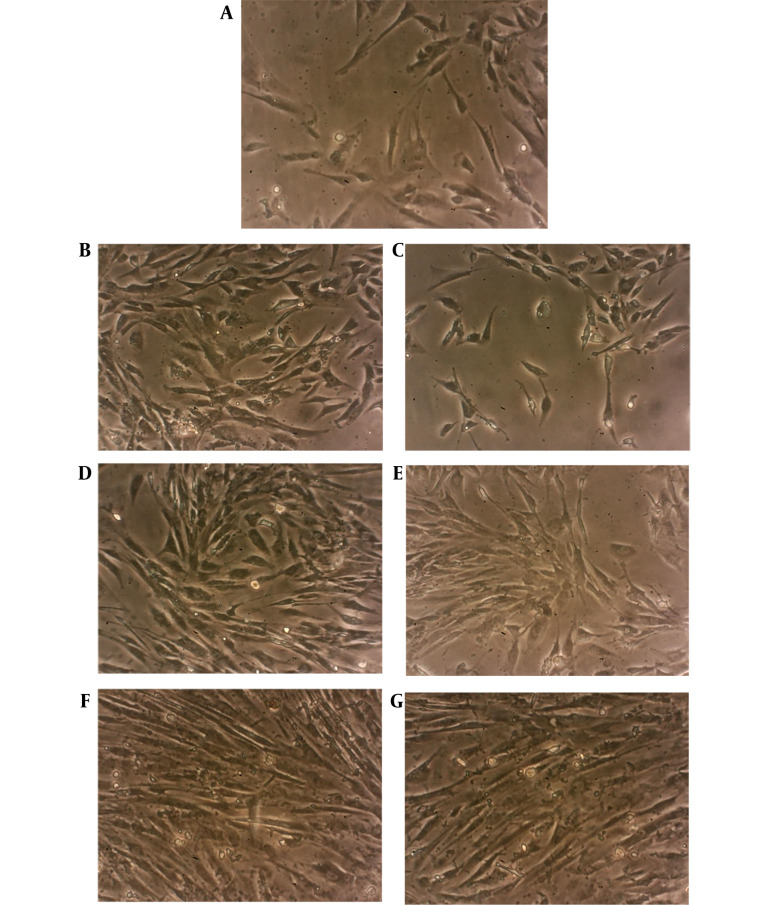
A, HCM cells in day 0 with elongated or rod-shaped appearance; B, HCM cells in the case group; and C, control group in day 1. Note to the higher cell density in the case cells due to augmented proliferation; D, HCM cells in the case group; and E, control group in day 3; F, HCM cells in the case group; and G, control group in day 6. Images were captured using inverted phase-contrast microscope (ECLIPSE, TS100, Nikon) equipped by a digital camera (magnification = 360X).

Human cardiomyocytes (HCMs; NCBI code: C599) were obtained from the local branch of the Pasteur Institute. These cells, derived from human heart tissue, were cultured in a specialized medium consisting of Ham’s F12 and Dulbecco’s modified Eagle medium (DMEM) at a 1:1 (v/v) ratio, supplemented with 10% fetal bovine serum (FBS), 5 µg/mL insulin, and 50 ng/mL basic fibroblast growth factor (bFGF) (Figure 1A). Upon reaching confluence, cells were detached using 0.05% trypsin-EDTA for 3 min in a humidified incubator maintained at 5% CO2. The resulting cell suspension was centrifuged at 1200 rpm for 10 min; the supernatant was discarded, and the cell pellet was resuspended in 2 mL of fresh culture medium. Subsequently, 1 mL of the cell suspension was seeded into a T25 culture flask containing 5 mL of culture medium. The flasks were incubated under the same conditions, and cells were allowed to proliferate until further experimental use.

### 3.2. Preparation of Cannabis Extract and Gas Chromatography-Mass Spectrometry

Flowering branches of *C. sativa* were obtained from a certified source and air-dried in a dark, non-humid environment. A hydroalcoholic extract was prepared using the percolation method with 70% ethanol as the extraction solvent. The solvent was completely removed using a rotary evaporator (IKA, Germany) at 50 rpm and 45°C. The resulting concentrate was further reduced under vacuum to obtain a viscous extract. The final preparation was transferred to glass containers and stored under refrigeration until use. A portion of the extract was analyzed by gas chromatography-mass spectrometry to identify its components.

Briefly, chemical profiling of the extract was performed using a 7890A gas chromatography system (Agilent Technologies) interfaced with a 7000 Triple Quadrupole mass spectrometer. Ionization was achieved by electron impact at an energy of 70 eV. Helium was used as the mobile phase at a constant flow rate of 1.2 mL/min. The injection port and transfer-line temperatures were set at 250°C and 280°C, respectively. Chromatographic separation was performed using a DB-1MS fused-silica capillary column (30 m × 0.25 mm i.d., 0.25 μm film thickness). The column oven temperature was programmed to increase from 60°C to 280°C at a ramp rate of 4°C per minute, followed by a 4-min isothermal hold. A 0.1 μL aliquot was introduced using split injection at a ratio of 1:30, and mass spectral data were collected over a scan range of 46 - 650 m/z. Compounds were identified by matching mass spectral profiles and retention indices with reference data from the Wiley and NIST libraries, as well as the database reported by Adams. Relative peak area percentages were calculated to enable quantitative comparison among samples. Quantitative composition was subsequently estimated using the instrument software based on the proportional contribution of each chromatographic peak to the total integrated area.

### 3.3. MTT Assay

To assess the effect of cannabis extract on cell viability and determine the optimal concentration for subsequent experiments, an MTT assay was performed. Human cardiomyocytes were seeded into three 96-well plates to evaluate the extract under three treatment regimens: (1) Single exposure for 1 day; (2) single exposure for 3 days; and (3) repeated exposure once daily for 3 consecutive days. After 24 h of incubation, varying concentrations of cannabis extract (50, 100, 500, 1000, 5000, and 10000 ng/mL) were added to each well in triplicate. Plates were incubated under standard culture conditions, and at the designated time points, the culture medium was removed and replaced with an MTT solution. After a 4-h incubation, plates were centrifuged, the supernatant was discarded, and dimethyl sulfoxide was added to dissolve the formazan crystals. Absorbance was measured at 570 nm using a spectrophotometer. The assay was performed in triplicate. Based on the results, 100 ng/mL was selected for downstream experiments because this concentration did not produce significant proliferative or inhibitory effects. Notably, this value approximates the reported blood concentration of cannabis in smokers (30).

### 3.4. Definition of Case and Control Groups

Two T25 culture flasks at passage 2 were prepared for the experimental setup. The stock cannabis extract solution (20 μg/mL) was diluted to a final concentration of 100 ng/mL and applied to the HCM culture designated as the case group. The control group consisted of HCM cultures treated with an equivalent volume of phosphate-buffered saline. Images were captured using an inverted microscope equipped with a digital camera. All experiments were performed in triplicate.

### 3.5. Growth Curve and Population Doubling Time

Human cardiomyocytes were seeded into two 18-well plates, one for the case group and the other for the control group. Twenty-four hours after seeding, three wells from each plate were selected for cell counting. Cells were detached using trypsin-EDTA, centrifuged, and the resulting pellet was resuspended in 1 mL of culture medium. Equal volumes of the suspension and trypan blue dye were mixed, and 2 μL of the mixture was loaded onto a Neubauer chamber under a coverslip. Cells were counted in four peripheral squares, and the mean cell number was recorded as day 1. This procedure was repeated daily until day 6. All measurements were conducted in triplicate.

### 3.6. Expression of Specific Cardiac Markers

Human cardiomyocytes were cultured in three T25 flasks for the case group, with each flask designated for analysis on days 1, 3, and 6. Corresponding flasks were prepared for the control group. At each time point, the culture medium was removed, and cells were detached using trypsin-EDTA. After centrifugation, total RNA was extracted using Rnx Plus Solution (CinnaGen, Iran). RNA quality and concentration were assessed using a NanoDrop 2000c spectrophotometer (Thermo Fisher Scientific, USA). Complementary DNA was synthesized from the RNA template using the AddScript cDNA Synthesis Kit (Addbio, South Korea). Quantitative real-time polymerase chain reaction was then performed using RealQ Plus 2× Master Mix Green (Ampliqon, Denmark) and specific primers on a StepOne system (Applied Biosystems). β2-microglobulin served as the endogenous control (Table 1). All reactions were run in technical triplicate, and the procedure was repeated three times.

**Table 1.** Primer Sequences Used in Real-Time Polymerase Chain Reaction

Marker	Forward (5’-->3’)	Reverse (5’-->3’)
**GATA4**	CCCAATCTCGTAGATATG	GCTGAGGCTTGATGAG
**Troponin T2**	AGAGGAGGACTGGAGAG	TCTTCTTCTTCATCTTCTTCTG
**Creatine kinase**	ATGACCACTTCCTGTTC	ATCTCCTCAATCTTCTGC
**β2-microglobulin**	TTCATCCATCCGACATT	CATCTTCAAACCTCCAT

### 3.7. Statistical Analysis

Continuous variables were expressed as the mean ± standard deviation. Depending on the data type, statistical comparisons were conducted using independent-samples t-tests or one-way analysis of variance. Analyses were performed using SPSS software version 16 (IBM Corp., USA). Gene expression levels were calculated using the 2^−ΔΔCt^ method. A P-value < 0.05 was considered statistically significant.

## 4. Results

### 4.1. Human Cardiomyocyte Culture

Human cardiomyocytes are among the most physically active cells in the body and exhibit contractions independent of nervous stimulation. At the start of culture, these micrometer-sized cells appeared in the flasks as binucleated, elongated, rod-shaped cells. After 24 h, no notable differences in HCM morphology were observed between the case and control groups. However, the case group showed higher cell density, indicating stimulated proliferation compared with the control group (Figure 1B and C). By day 3, the difference in cell density between the groups became less pronounced, likely because of accelerated proliferation in the control group along with a mild increase in the case group. Morphologically, the cells remained similar in both groups (Figure 1D and E). On day 6, limited space in the culture flasks resulted in confluent growth in both groups, with cells exhibiting similar shapes (Figure 1F and G).

**Figure 2.**
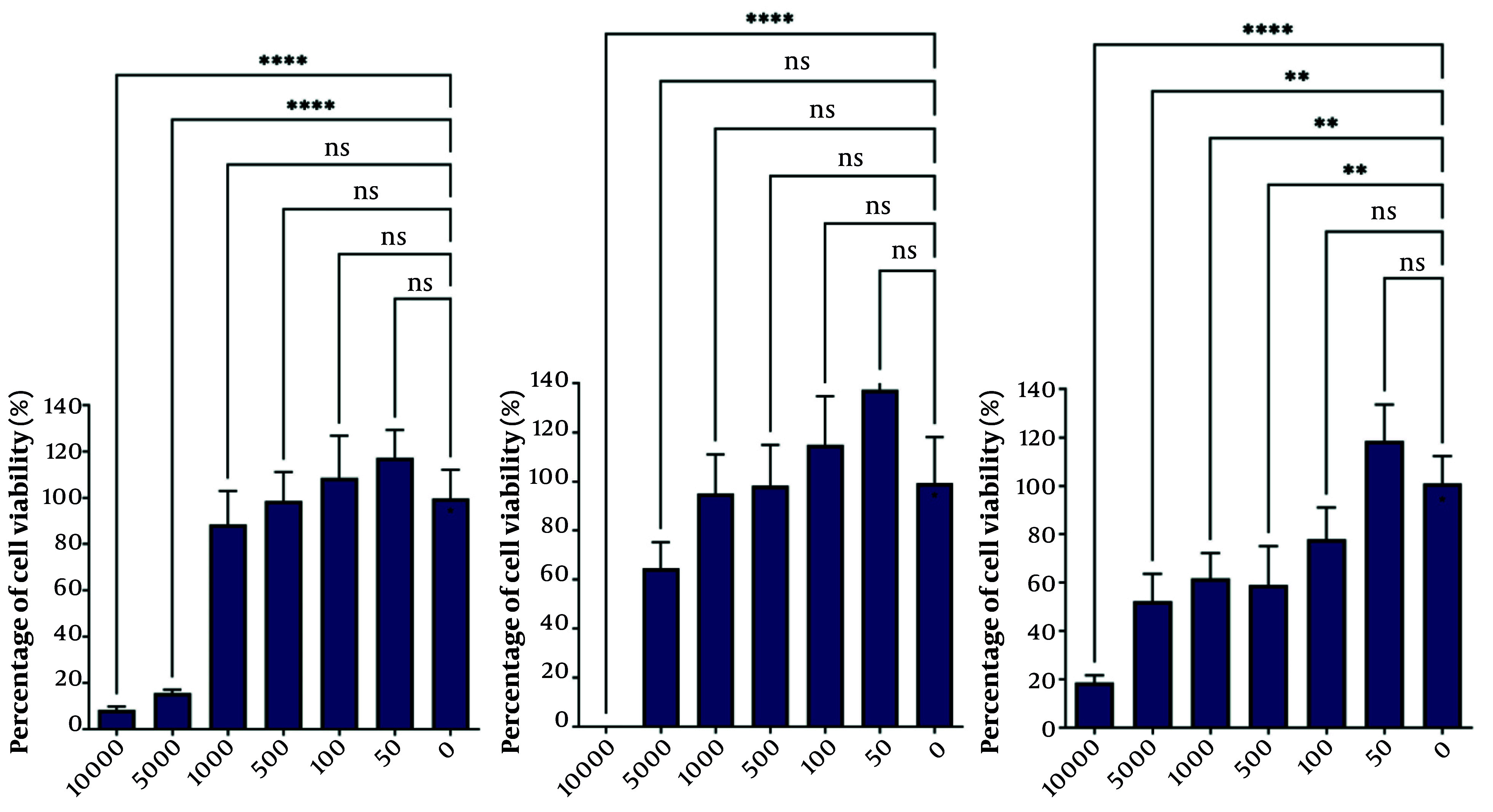
MTT assay shows HCM viability after exposure to different concentrations of cannabis extract. Left: One time exposure during one day, Center: One time exposure during three days, and Right: Three times exposure during three days. X axis shows concentration of Cannabis sativa extract in ng/mL and Y axis shows percentage of cell viability (%) (ns, non-significant; Statistical significance was P-value < 0.05; ** P-value < 0.005 and **** P-value < 0.00005).

The viability of HCMs exposed to varying concentrations of cannabis was assessed using an MTT assay under three different conditions (Figure 2). Concentrations of 50, 100, 500, 1000, 5000, and 10000 ng/mL of the extract were prepared and applied to HCMs under three conditions: (1) One-time exposure for 1 day, (2) One-time exposure for 3 days, and (3) Three exposures over 3 days. In the first condition, the lowest viability was observed after exposure of HCMs to 5000 and 10000 ng/mL (P < 0.05), whereas in the second condition, 10000 ng/mL was the only concentration that caused statistically significant growth inhibition (P < 0.05). In the third condition, all concentrations except 50 and 100 ng/mL inhibited HCM growth (P < 0.05).

### 4.2. Gas Chromatography-Mass Spectrometry

Analysis of the extract identified several components (Table 2).

**Table 2.** Components of the Cannabis Extract Identified by Gas Chromatography-Mass Spectrometry

Item	Component	Percent	Item	Component	Percent	Item	Component	Percent
1	Pinene (a-)	3.818	11	Pinene hydrate (trans)	0.471	21	Selinene (a-)	2.451
2	Camphene	0.391	12	Isoborneol	0.364	22	Bisabolene (z-a)	1.141
3	Pinene (β-)	2.148	13	Terpineol (a-)	0.835	23	Alaskene (a-)	0.828
4	Myrcene	7.085	14	Ylangene (a-)	0.4	24	Cadinene (ð-)	1.231
5	Limonene	10.4	15	CBD	0.3363	25	THC	0.229
6	Ocimene (E-B)	0.572	16	Caryophyllene (E-)	22.877	26	Zonarene	10.001
7	Terpinene (y-)	0.421	17	Bergamotene (a-trans)	1.04	27	Selina-3,7 (11)-diene	10.734
8	Mentha-2,4 (8)-diene (ρ)	0.89	18	Humulene (a-)	10.745	28	Nerolidol (E-)	1.224
9	Linalool	3.801	19	Chamigrene (β-)	0.405	29	Maaliol	0.784
10	Fenchol (endo-)	1.279	20	Selinene (β-)	2.75	30	Caryophyllene oxide	0.325

### 4.3. Growth Curve and Population Doubling Time

**Figure 3.**
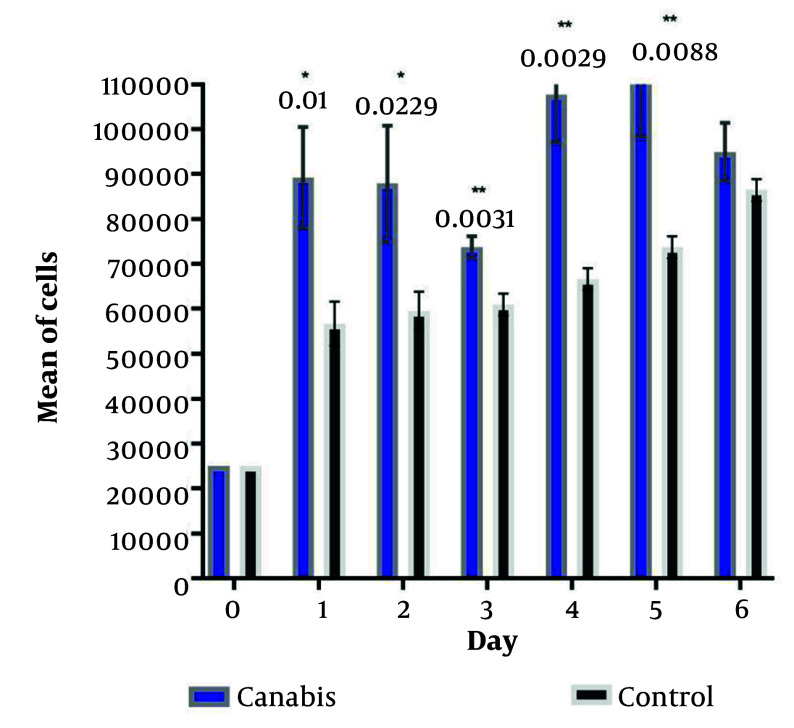
Growth curve of the case and control groups during six days. In all time points, number of cells in the case group was higher (* P-value < 0.05; ** P-value < 0.01; Statistical significance was P value < 0.05).

**Figure 4.**
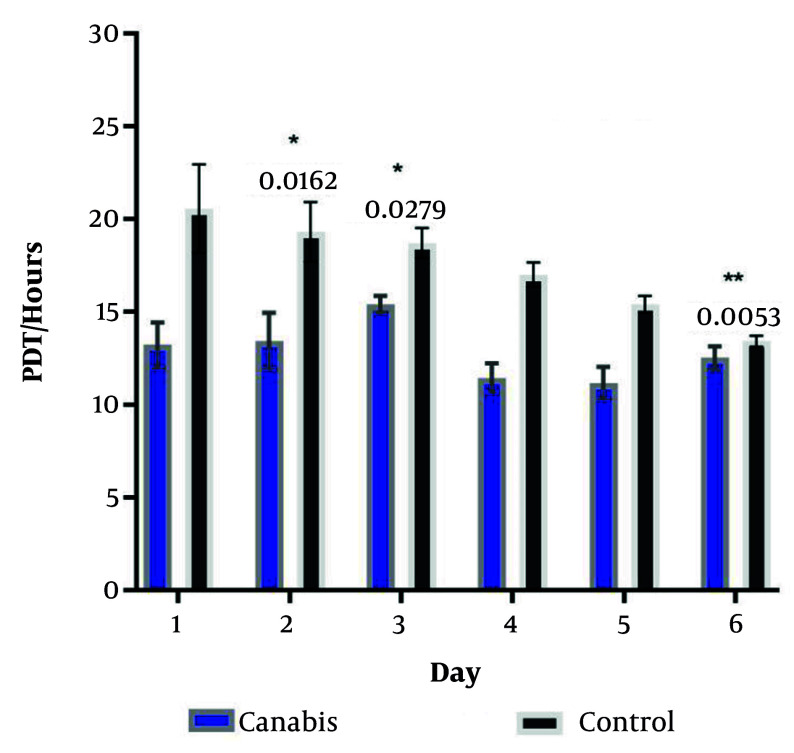
Population doubling time of HCM in the case and control groups in six consecutive days (* P-value < 0.05; ** P-value < 0.01; Statistical significance was P-value < 0.05).

As illustrated in Figure 3, the number of cells in the case group was generally higher than that in the control group over the 6-day period. Although cell counts in the case group decreased at two time points (day 3 and day 6) compared with the previous day, they remained higher than those in the control group at both time points. Consistent with the growth-curve results, the population doubling time (PDT) was generally lower in the case group than in the control group throughout the 6 days. Specifically, on day 6, the PDT was 74.79 h for the case group and 80.45 h for the control group. Notably, the smallest differences in PDT between the groups were observed on days 3 and 6 (Figure 4).

### 4.4. Expression of Specific Cardiac Markers

GATA4, troponin, and cardiac creatine kinase are key markers specifically expressed by cardiomyocytes. GATA4, an early-stage marker, showed a decreasing expression trend in the case group compared with the control group over 6 days. Its expression differed significantly between day 1 and both days 3 and 6, whereas the difference between days 3 and 6 was not significant. A similar decreasing trend was observed for troponin expression over the 6-day period. Troponin levels in the case group were significantly higher on day 1 but decreased on days 3 and 6. In contrast, creatine kinase, a late-stage marker, peaked in expression on day 3, with the lowest expression observed on day 1. The increase on day 3 was statistically significant compared with both day 1 and day 6, whereas no significant difference was found between day 1 and day 6 (Table 3).

**Table 3.** Expression Ratio of Cardiac-Specific Markers in the Treatment Group vs. Control Group ^a^

Marker	Day 1	Day 3	Day 6	P-value	P-value Day 1 vs. 3	P-value Day 1 vs. 6	P-value Day 3 vs. 6
**GATA4**	7.976 ± 1.068	1.484 ± 0.643	0.110 ± 0.251	< 0.001	< 0.001	< 0.001	0.131
**Troponin**	3.529 ± 0.854	1.137 ± 0.927	0.085 ± 0.694	0.006	0.028	0.005	0.334
**Creatine kinase**	0.001 ± 0.0008	1.221 ± 0.909	0.136 ± 0.262	0.022	0.028	0.935	0.048

^a^ Values are fold changes in the treatment group vs. the control group derived from 2^−ΔΔCt^ method analysis.

## 5. Discussion

To the best of our knowledge, this is the first study to investigate the impact of *C. sativa* extract on the biological activity of human cardiomyocytes. Over the 6-day period, no significant morphological changes were observed in cardiomyocytes exposed to cannabis extract compared with control cells. However, cell proliferation appeared to be enhanced, particularly during the first 3 days. Furthermore, the expression of cardiac-specific markers increased in the treatment group during this period. Overall, our findings indicate that the biological activity of human cardiomyocytes is notably enhanced during the early days of exposure. However, this effect diminished thereafter, possibly because of a reduced influence of the extract as cell numbers increased and the cells adapted to the conditioned medium by day 6.

Over recent decades, the catalog of naturally occurring constituents identified or isolated from *C. sativa* has expanded steadily, reaching 545 distinct compounds (31). Extracts derived from *C. sativa* have been found to possess pro-healing activity, plausibly attributable to the anti-inflammatory and antioxidant actions of their predominant bioactive compounds; these effects have also been demonstrated in in vitro cellular models (32). In particular, all types of cannabinoids substantially affect the cardiovascular system (23, 33, 34). Numerous studies have linked cannabis use to arrhythmia, MI, and acute coronary syndrome (24-26, 35).

The primary factor underlying cannabis-related cardiovascular complications is the high concentration of THC in the plant (36). The formation of stress fibers and cell elongation after treatment with primary metabolites of THC has been reported, indicating cytoskeletal remodeling and cell polarization. In H9c2 cardiomyocytes, these THC metabolites also downregulate β-catenin (37). Further evidence of the harmful effects of THC on cell integrity and structure comes from studies in which high-dose THC metabolite treatment induced pronounced toxicity, characterized by irregular nuclei, cytoskeletal degradation, and membrane perforations (37). Conversely, pretreatment with an ultra-low dose of THC has been shown to significantly protect the heart against ischemic injury, as evidenced by reduced troponin levels and smaller infarct size (38).

Another important component of extracts derived from *C. sativa* is CBD, which has a broad spectrum of pharmacological activities, including neuroprotective, anti-inflammatory, antineoplastic, and analgesic effects (39, 40). Accumulating experimental and clinical evidence further supports its role as a protective agent in cardiovascular pathology (41). A recent investigation showed that CBD treatment significantly enhanced myocardial regenerative capacity, reduced infarct area, and improved functional recovery of cardiac performance after MI (42). The study further demonstrated that CBD stimulated the proliferation of neonatal cardiomyocytes, consistent with our findings. In fact, CBD exposure was associated with marked suppression of miR-143 - 3p. Moreover, CBD upregulated the expression of Yes-associated protein and catenin delta 1, both of which were identified as downstream targets of miR-143 - 3p (42). Cannabidiol mitigates the adverse effects of high glucose on primary HCMs by reducing the production of reactive oxygen and nitrogen species, inhibiting nuclear factor-κB activation, and preventing cell death (43). Cannabidiol also inhibits vascular smooth muscle cell migration and proliferation induced by growth factors (44) and reduces the inflammatory response caused by elevated glucose levels in endothelial cells within the human coronary artery (45).

In addition to THC and CBD, *C. sativa* contains a range of secondary metabolites (46). Friedelin has been documented to exert anti-inflammatory, antioxidant, antipyretic, anticarcinogenic, and antitumor effects (32). Epi-friedelanol has been reported to demonstrate notable pharmacological properties, including anticancer activity (47), suppression of inflammatory responses (48), and protective effects against cellular senescence (49). Sterols are also associated with antihypercholesterolemic and antitumor activities (50). β-sitosterol exhibits pronounced anti-inflammatory potential (51), as evidenced by its ability to reduce peptidoglycan-induced secretion of pro-inflammatory mediators in keratinocytes and macrophages, downregulate NLRP3 inflammasome expression, and suppress both caspase-1 activation and nuclear factor-κB signaling (32).

Recent independent investigations have shown that friedelin engages anti-inflammatory pathways in murine models. This protective effect is associated with reduced accumulation of the pro-inflammatory cytokines tumor necrosis factor-α, interleukin (IL)-1, and IL-6, as well as suppression of autophagic processes mediated through modulation of the AMPK-mTOR signaling axis (52). The attenuation of IL-6 production appears to be mediated by activation of the miR-146a/IRAK-1 regulatory pathway, whereby upregulation of miR-146a is concomitant with reduced IRAK-1 expression and contributes to the overall anti-inflammatory profile of *C. sativa* (32). From a pharmacological perspective, this mechanism may be clinically relevant because it suggests potential synergistic interactions with therapeutics that directly target IL-6 signaling, including monoclonal antibodies against the IL-6 receptor, such as sarilumab and tocilizumab, or IL-6 itself (53).

Overall, marijuana exposure induces severe structural changes in the heart, an organ with limited regenerative capacity. These alterations include cardiac hypertrophy, increased deposition of extracellular matrix proteins leading to reduced contractility, and, with chronic repeated use, cell death and an irreversible decline in cardiac function (37). Nonetheless, some indirect benefits associated with cannabis use, such as reduced rates of other types of smoking (54), a lower prevalence of obesity and diabetes mellitus, decreased fasting insulin levels and insulin resistance, and reduced waist circumference (55), should not overshadow its detrimental effects on the cardiovascular system.

This study has several limitations. First, monitoring changes in a broader range of markers would have strengthened the findings. In addition, functional assays in cardiomyocytes from the case group were not feasible in this study. In future research, we plan to evaluate the effects of cannabis extract on cardiomyocytes over a longer duration to better simulate chronic use. Moreover, animal model studies will help provide further insights into the cardiovascular effects of cannabis.

In conclusion, the extract derived from *C. sativa* altered growth kinetics and the expression of cardiac-specific markers in human cardiomyocytes. These findings warrant consideration in the context of cardiovascular health because such effects may impair normal cardiac function, an issue of particular concern given the rising use of cannabis, especially among young individuals.

## Data Availability

The dataset presented in the study is available on request from the corresponding author during submission or after publication.
